# Linking diabetes to worsening knee osteoarthritis symptoms and disability: evidence from the osteoarthritis initiative

**DOI:** 10.3389/fmed.2026.1759342

**Published:** 2026-02-18

**Authors:** Aqeel M. Alenazi, Sultan A. Alanazi, Abdullah M. Alqthami, Mohammed K. Alanazi, Bader A. Alqahtani, Norah A. Alhwoaimel, Ahmad D. Alanazi, Sattam M. Almutairi, Mohammed S. Alghamdi, Yasir S. Alshehri, Vishal Vennu, Saad M. Bindawas

**Affiliations:** 1 Department of Health and Rehabilitation Sciences, College of Applied Medical Sciences, Prince Sattam Bin Abdulaziz University, Alkharj, Saudi Arabia; 2 Department of Physical Therapy and Health Rehabilitation, College of Applied Medical Sciences, Majmaah University, Al-Majmaah, Saudi Arabia; 3 Department of Rehabilitation, King Khalid Hospital at Alkharj, Alkharj, Saudi Arabia; 4 Comprehensive Rehabilitation Center, Ministry of Human Resources and Social Development, Arar, Saudi Arabia; 5 Department of Physical Therapy, College of Applied Medical Sciences, Qassim University, Buraydah, Saudi Arabia; 6 Department of Medical Rehabilitation Sciences, College of Applied Medical Sciences, Umm Al-Qura University, Makkah, Saudi Arabia; 7 Department of Physical Therapy, College of Medical Rehabilitation Sciences, Taibah University, Madinah, Saudi Arabia; 8 Department of Rehabilitation Sciences, King Saud University, Riyadh, Saudi Arabia

**Keywords:** arthritis, diabetics, disability, pain intensity, physical functions

## Abstract

**Objective:**

To examine the impact of baseline diabetes mellitus (DM) on longitudinal knee symptoms and disability in participants with knee osteoarthritis (OA).

**Methods:**

This was a secondary analysis using publicly available data from a longitudinal study design (Osteoarthritis Initiative). Data from 4,796 participants (45–79 years of age) were obtained, and only participants with grade ≥2 in either knee, using the Kellgren and Lawrence grading at baseline, were included in the current analysis. Based on self-reported DM, the participants were categorized into two groups: knee OA and DM or knee OA only. Symptoms and disability were measured using the Western Ontario and McMaster Universities Arthritis Index (WOMAC) scale and pain severity across 7- and 30-days at seven visits from baseline to 96 months of follow up.

**Results:**

A total of 2,486 participants were included and categorized into knee OA and DM (*n* = 221) and knee OA only (*n* = 2,265). The longitudinal analyses results (*n* = 2,483) showed that participants with knee OA and DM had significantly increased knee symptoms over time using the WOMAC total score [beta (*B*) = 3.20, *p* = 0.004], WOMAC pain subscale (*B* = 0.71, *p* = 0.003), WOMAC stiffness subscale (*B* = 0.22, *p* = 0.036), and WOMAC disability subscale (*B* = 2.26, *p* = 0.005) compared with participants with knee OA only. Knee OA and DM was not associated with increased knee pain severity over a 7-day period (*B* = 0.28, *p* = 0.10) and over a 30-day period (*B* = 0.20, *p* = 0.23) when compared to knee OA only.

**Conclusion:**

This longitudinal cohort study provides evidence supporting the association between baseline DM and increased knee symptoms and disability among individuals with knee OA.

## Highlights

What is known

- Diabetes has been linked with knee osteoarthritis and knee pain using cross-sectional studies.

What is new

- Baseline diabetes was associated with more knee related symptoms overtime across 96 months of follow up in people with knee osteoarthritis.
- Diabetes was associated with a longitudinal increase in knee pain, stiffness, and disability in people with knee osteoarthritis.

## Introduction

Osteoarthritis (OA) is a debilitating ailment impacting individuals globally, with an estimated frequency of 16% among the general population (1–4). OA commonly affects various joints of the body, including the knees, hips, hands, and spine (5). The predominant symptoms associated with OA are pain, stiffness, and physical limitations, which substantially impact function and often necessitate treatment to improve the quality of life. These symptoms and physical limitations can be influenced by a wide range of factors including age, sex, obesity, and the presence of comorbidities such as diabetes mellitus (DM) (6–11).

DM is a prevalent metabolic disorder that affects approximately 11% of the overall population and causes various complications (e.g., hyperglycemia) (12). Hyperglycemia, a prevalent complication of DM, has the potential to affect the health of the joints and bones. Elevated glucose levels in individuals with DM can trigger chronic systemic inflammation, which, in turn, induces systemic alterations in various systems and joints, leading to further complications (13). Furthermore, advanced glycation end products (AGEs) secondary to hyperglycemia accumulate throughout the body, including in joints. This accumulation can induce cartilage stiffness and increase bone fragility, thereby contributing to joint degeneration (14). Recent evidence has indicated potential mechanisms for DM on OA progression using animal model including activation of inflammatory markers (15, 16). Therefore, DM is associated with chronic musculoskeletal conditions, including OA, which can exacerbate symptoms and disability.

Research has explored the correlation between OA and DM in terms of prevalence, progression, symptoms, and physical function (6, 17–24). These studies have identified a strong association between OA and DM. Other studies found that DM is an independent risk factor for OA progression, leading to joint replacement (17–22). In terms of symptoms, the previous studies have linked DM to greater pain intensity in patients (9, 11, 17, 25–34). However, research on the impact of DM on physical functions and disability is limited (29, 35–39). Prior research has mainly focused on the influence of DM on gait speed or limitation, with muscle strength being the main outcome (8, 29, 38, 39). As DM is associated with declined gait speed and muscle strength among individuals with OA (29, 35–39), potential impacts on disability should be examined. Most previous studies assessed the effect of DM on pain and physical function in the OA population using a cross-sectional design (9–11, 29, 30). These studies shared common limitations, including the lack of control for important covariates, the use of different OA sites, and the lack of a longitudinal design. A recent longitudinal study examined metabolic syndrome and its components at baseline with the structural progression of knee OA over 5 years (40). This study found that increased glucose levels at baseline were associated with osteophyte formation during the follow up period (40). However, this study included only women and did not focus on knee symptoms. Consequently, it is critical to investigate how DM affects symptoms and disability in individuals with knee OA.

DM is considered a modifiable risk factor that requires tailored interventions targeting systemic factors rather than localized areas of the body in patients with knee OA. Therefore, examining DM in relation to knee OA symptoms and disabilities is critical. A 2021 study showed that a multidisciplinary lifestyle program, including nutrition, physical activity, and stress management for individuals with metabolic diseases, such as DM and knee OA, showed better improvements in outcomes, including pain and disability (41). This study indicated that different multidisciplinary approaches should be implemented for participants with knee OA and DM, targeting holistic bodies and systems. Thus, additional investigations are required to comprehensively understand how DM affects knee symptoms and disability in individuals with knee OA, and to establish appropriate interventional approaches. Therefore, this study aimed to examine the impact of baseline DM on longitudinal knee symptoms and disability in people with knee OA. We hypothesized that baseline DM would be related to an increase in knee symptoms, including pain, stiffness, and disability, over time in this population.

## Methods

This secondary analysis used longitudinal data from publicly available database, the Osteoarthritis Initiative (OAI), a cohort study that focused on understanding and managing the onset and progression of knee OA. A comprehensive description of the OAI study is available at https://nda.nih.gov/oai/. The OAI involved a diverse group of 4,796 participants between the ages of 45 and 79 years who either had knee OA or were at a high risk of developing it. High-risk individuals were those who did not have knee OA at the beginning of the study but were susceptible to developing it during the research. All participants completed seven visits over 96 months, during which they underwent biospecimen investigation, joint imaging, and clinical evaluation.

The study was performed in accordance with the 1964 Declaration of Helsinki and subsequent revisions. Informed consent was obtained from each participant prior to their participation. The research protocol was approved by the University of California, San Francisco, and its affiliates (approval number: FWA00000068), in addition to approval from all four clinical sites.

### Cohort selection

For the current study, only participants with knee OA were selected based on the baseline Kellgren and Lawrence (KL) grades ≥2. All participants were classified into two groups based on baseline self-reported DM: knee OA and DM (*n* = 221) and knee OA only (*n* = 2,265). Previous evidence has showed good validity and reliability using the Charlson Comorbidity Index for self-reported DM (42, 43). Data on outcome measures and related factors were collected across seven time points: baseline and 12, 24, 36, 48, 72, and 96 months.

### Outcome measures

Knee symptoms and disability were the primary outcome measures for the current study. Knee symptoms and disability were assessed using the 24-item scale known as the Western Ontario and McMaster Universities Arthritis Index (WOMAC), which includes three subscales: total pain, stiffness, and disability (44). Additionally, knee pain severity was evaluated over two different durations, namely, 7 and 30 days.

The total WOMAC score was measured at seven visits from baseline to 96 months (44). WOMAC scale has been validated in previous research with good sensitivity for pain and disability (45, 46). The WOMAC total score includes the overall symptoms and knee function. The WOMAC total score includes the sum of three subscales: pain, stiffness, and disability. The participants were asked to rate their activities according to the difficulty of using none, slight, moderate, severe, or extreme. The WOMAC total score ranges from 0 to 96, with higher scores indicating more symptoms and disability.

The WOMAC subscale for pain includes five items: walking, climbing stairs, nocturnal, resting, and weight-bearing. Participants were asked to rate their activities according to their level of difficulty. The scoring system used a 0–4 scale, where 0 indicated none, 1 indicated mild, 2 indicated moderate, 3 indicated severe, and 4 indicated extreme. Scores range from 0 to 20, with higher scores indicating worse pain.

The WOMAC subscale for stiffness included two items (morning stiffness and stiffness later in the day). The participants were asked to rate the difficulty according to their stiffness. The scoring system used a 0–4 scale, where 0 indicated none, 1 indicated mild, 2 indicated moderate, 3 indicated severe, and 4 indicated extreme. The scores ranged from 0 to 8, with higher scores indicating greater stiffness.

The WOMAC subscale for disability was measured at seven visits from baseline to 96 months (44). WOMAC disability subscale includes 17 self-reported items related to physical functions and activities of daily living, such as walking, shopping, and bathing. The scoring system used a 0–4 scale, where 0 indicated none, 1 indicated mild, 2 indicated moderate, 3 indicated severe, and 4 indicated extreme. The WOMAC disability subscale ranges from to 0–68 with higher scores indicating greater disability and functional limitations. This scale and its subscales were used for the right and left knees, and the knee that experienced the most symptoms was selected for the present analysis.

Pain intensity was measured at 7- and 30-days (47) to examine the short- and long-term effects of DM on pain. This scale was measured during seven visits from baseline to 96 months. Participants rated their pain in the past 7 days and 30 days using a numeric rating scale (0 = no pain to 10 = severe pain) for each knee. For this investigation, the knee with the most severe symptoms was selected.

### Covariates

Covariates were demographic factors [age, sex, race, education, and body mass index (BMI)] and clinical variables [depressive symptoms using the Center for Epidemiologic Studies for Depression (CES-D) (48) and physical activity level using the Physical Activity Scale for the Elderly (PASE)] (49). These covariates have been adjusted for in previous studies (50). Age (years) was used as a time-dependent covariate from baseline to 96 months of follow-up. Race was coded as binary (White vs. Others, such as Black/Asian/Other non-white people), as the majority of the samples were white. Educational level was coded into six levels including less than high school (reference category), high school graduate, some college, college graduate, some graduate school, and graduate degree. BMI was used as a time-dependent covariate from baseline to 96 months follow up. Depressive symptoms were assessed using the CES-D scale, a self-reported 20-item scale, and a cut-off score of 16 was used to categorize participants with depressive symptoms (48). Depressive symptoms were used as time-dependent covariates from baseline to 96 months of follow-up. The PASE was used as a time-dependent covariate from baseline to 96 months follow up (49).

### Data analysis

Data were examined by analyzing continuous and categorical variables to provide descriptive statistics regarding baseline demographics and clinical variables. Differences between participants with knee OA and DM and those with knee OA only were assessed using the *t*-test and chi-squared test for continuous and categorical variables, respectively.

To assess the impact of DM on knee symptoms and disability, six models were created using linear regression analysis with Generalized Estimating Equations (GEE). DM was included as a predictive variable, with the reference category being participants without DM. The dependent variables were separately entered for each model, including the WOMAC total, WOMAC pain, WOMAC stiffness, WOMAC disability, and knee pain severity at 7- and 30-days. The model accounted for potential confounding factors including age, sex, race, educational level, BMI, CES-D, and PASE. The significance level for all analyses was established at 0.05. IBM SPSS for Mac version 25.0 (SPSS Inc., Chicago, IL, USA) was used for data analysis.

## Results

**Figure 1:**
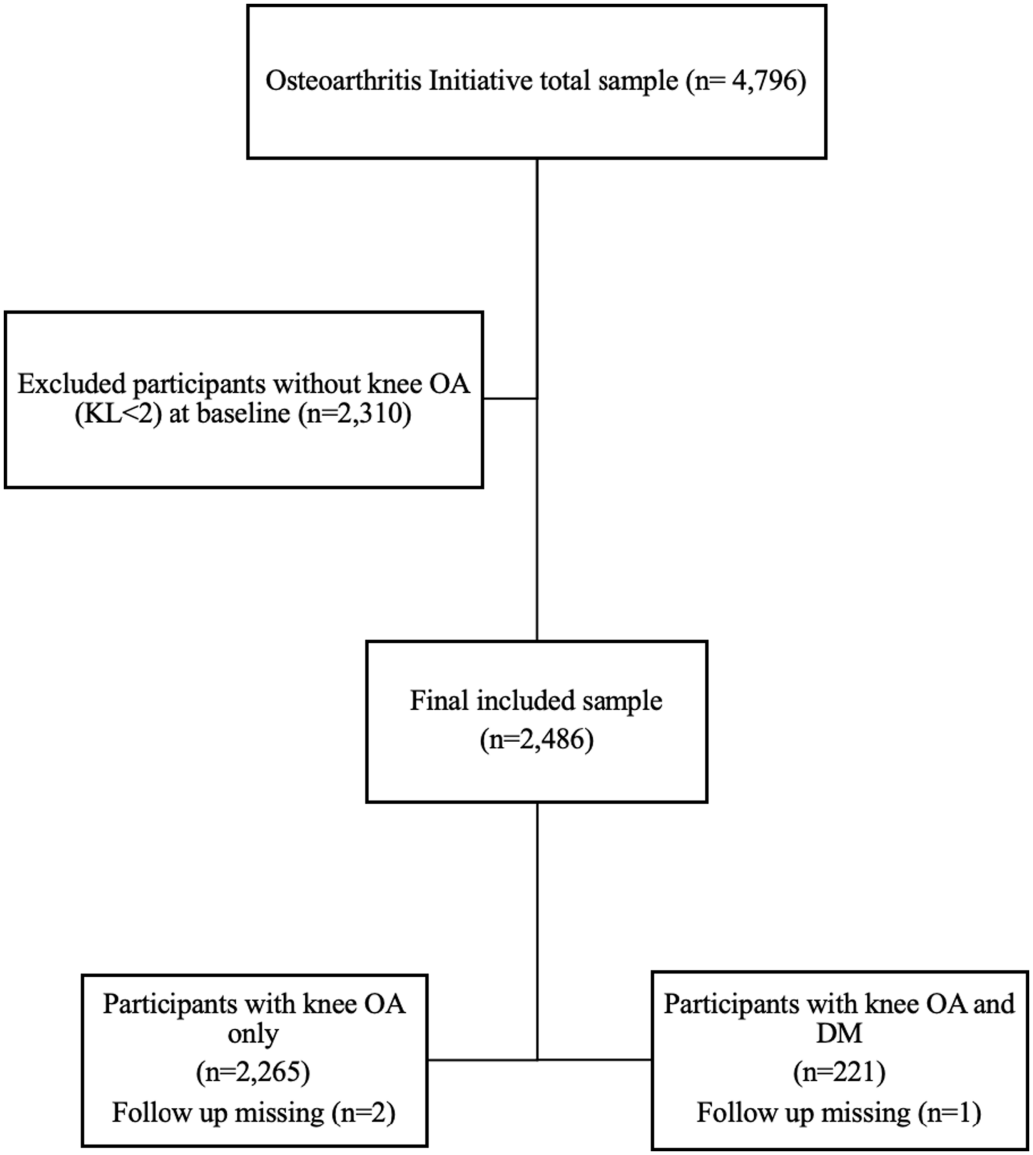
Flow chart of the participants selection.

A total of 2,486 participants with knee OA at baseline (KL grade ≥2) based on the OAI were included. Figure 1 shows the selection process for participants from the OAI dataset. Participants with a KL grade of 0 or 1 were excluded. The participants were divided into two groups: those with knee OA and DM (*n* = 221) and those with knee OA only (*n* = 2,265). Table 1 shows baseline comparisons between the groups in terms of demographics and clinical variables. Age, race, BMI, educational level, depressive symptoms, PASE score, WOMAC total score, and pain, stiffness, and disability subscales were significantly different between the groups. Knee pain severity at 7 and 30 days was significantly higher in people with knee OA and DM than in those with knee OA alone. A total of 3 participants lost to follow up during the subsequent visits and the total sample for the longitudinal analysis was 2,483 as shown in Table 2.

**Table 1:** Baseline and clinical characteristics.

Factors	Knee OA only *N* = 2,265	DM and knee OA *N* = 221	*p-*value
Age, years (mean ± SD)	62.38 ± 9	64.11 ± 8	0.006
Sex, females, *n* (% within DM)	1,309 (57.8)	129 (58.4)	0.887
Race, white, *n* (% within DM)	1799 (79.4)	131 (59.3)	<0.001
BMI, Kg/m^2^ (mean ± SD)	29.33 ± 4.7	31.96 ± 4.6	<0.001
Education, *n* (% within DM)			<0.001
Less than high school	82 (3.6)	14 (6.3)	
High school graduate	277 (12.2)	52 (23.5)	
Some college	541 (23.9)	78 (35.3)	
College graduate	481 (21.2)	35 (15.8)	
Some graduate school	182 (8.0)	13 (5.9)	
Graduate degree	701 (31.0)	29 (13.1)	
Depression, *n* (% within DM)	213 (9.4)	32 (14.8)	0.017
PASE (mean ± SD)	158 ± 81	130 ± 70	<0.001
WOMAC total (mean ± SD)	18.33 ± 18.21	26.05 ± 20.07	<0.001
WOMAC pain (mean ± SD)	3.91 ± 3.93	5.63 ± 4.61	<0.001
WOMAC stuffiness (mean ± SD)	2.25 ± 1.80	2.88 ± 1.89	<0.001
WOMAC disability (mean ± SD)	12.38 ± 12.46	17.73 ± 14.39	<0.001
Pain severity over 7 days (mean ± SD)	3.74 ± 2.72	4.61 ± 2.95	<0.001
Pain severity over 30 days (mean ± SD)	3.95 ± 2.85	4.73 ± 3.18	0.001

OA, osteoarthritis; *N*, number; DM, diabetes mellitus; SD, standard deviation; BMI, body mass index; PASE, Physical Activity for Elderly Scale; WOMAC, Western Ontario and McMaster Universities Arthritis Index (WOMAC).

**Table 2:** GEE with linear regression for the relationship between baseline DM and overtime pain severity scores and WOMAC scores.

Factors	Model (*n* = 2,483)		
	*B*	SE	*p*-value
WOMAC total	3.20	1.12	0.004
WOMAC pain	0.71	0.24	0.003
WOMAC stuffiness	0.22	0.11	0.036
WOMAC disability	2.26	0.81	0.005
Pain severity over 7 days	0.28	0.17	0.10
Pain severity over 30 days	0.20	0.17	0.23

*B*, beta (unstandardized coefficients); SE, standard error.

All models were adjusted for age, sex, race, education, body mass index, depressive symptoms, and physical activity level.

References for covariates categorical variables were knee OA without DM, male sex, white race, and high school/less education.

The results of the GEE using linear regression analysis examining the association between baseline knee OA and DM compared to knee OA only with longitudinal knee symptoms and disability using the WOMAC total score, WOMAC pain, WOMAC stiffness, WOMAC disability, and knee pain severity over 7 and 30 days are shown in Table 2. The results revealed that participants with knee OA and DM had significantly increased overtime knee symptoms using WOMAC total score [Beta (*B*) = 3.20, *p* = 0.004], increased WOMAC pain subscale (*B* = 0.71, *p* = 0.003), increased WOMAC stiffness subscale (*B* = 0.22, *p* = 0.036), and increased WOMAC disability subscale (*B* = 2.26, *p* = 0.005) when compared to participants with knee OA only after controlling for covariates including age, sex, race, education, BMI, depressive symptoms using CES-D, and physical activity level using PASE. However, knee OA and DM was not significantly associated with overtime increased knee pain severity over 7 days (*B* = 0.28, *p* = 0.010) and over 30 days (*B* = 0.20, *p* = 0.23) when compared to knee OA only after controlling for covariates.

## Discussion

The present investigation revealed that participants with baseline knee OA and DM exhibited a progressive increase in knee symptoms, including pain, stiffness, and disability scores (assessed using the WOMAC scale) when compared to knee OA only. This association was evident after controlling for covariates, such as age, sex, race, BMI, physical activity level, and depressive symptoms. However, knee OA and DM did not exhibit an association with overtime knee pain severity when assessed using a numeric rating scale over 7-days and over a 30-day period.

Our findings were consistent with earlier reports assessing the relationship between DM and knee pain in patients with knee OA (9, 11, 17, 25–34). However, most previous studies were either cross-sectional or had a limited follow-up period (9, 11, 17, 25–34). Our findings were consistent between the WOMAC subscale except for knee pain severity over 7-days and over 30 days, which was not significantly associated with DM in this population. This could be attributed to the control of covariates that might affect the association, as the baseline knee pain severity over 7-days and over 30 days were significantly greater in patients with knee OA and DM than in participants with knee OA only. This discrepancy between the WOMAC and numeric rating scales may also be attributed to the distinct temporal dynamics that influence pain perception. Moreover, individual variations in DM management and overall health status within the study population may further contribute to the differing associations over different timeframes, emphasizing the need for comprehensive assessments to elucidate the temporal nuances of the DM-knee pain relationship. Additional research is required to explore the association between DM using objective metrics, such as glucose and A1c levels, and knee symptoms in this population.

The relationship between DM and increased disability in individuals with knee OA is of particular significance to clinicians because both conditions have a considerable impact on an individual’s quality of life. Our results are consistent with those of previous studies that found a higher burden of limited physical function among individuals with comorbid DM and OA (29, 35–39). These findings suggest that DM may contribute to the worsening of knee OA symptoms and functional limitations—leading to a poor quality of life. However, these previous studies did not examine the association between DM and knee-related disability over time and focused only on walking speed and muscle strength (29, 35–39).

One possible mechanism explaining this association is the potentially detrimental effect of hyperglycemia on the joint health of patients with knee OA. Hyperglycemia can lead to chronic systemic inflammation, which may induce changes in various body organs as well as within the joints. Consequently, the joint structures may become compromised, leading to increased disability. Previous studies have identified a negative impact of DM on joint pain (9, 11, 17, 25–34)—leading to greater disability and limited physical function. A study conducted in 2020 found that DM was related to pain during ambulation in individuals with knee OA (29). Future research should investigate the potential mechanisms associated with disability in this population, such as hyperglycemia and glucose levels.

Important clinical implications can be drawn from this study to support clinical decision-making processes. Clinicians should comprehensively establish interventional strategies for individuals with DM and knee OA that focus on glycemic control. A multidisciplinary approach involving healthcare providers, including rheumatologists, orthopedic specialists, and endocrinologists, may be necessary to optimize treatment outcomes, improve physical function, and prevent or delay the incidence of disability. Additionally, lifestyle modifications targeting weight management, and the promotion of physical activity should be emphasized in this population to alleviate symptoms and potentially slow the progression of disability.

The strengths of the current study include its design and control of possible covariates that may influence the relationship between DM and knee OA in terms of knee symptoms and disability. Our study investigated the association between DM and knee symptoms using large-scale longitudinal data at 96 months of follow up. Another strength of this study was the use of more comprehensive knee symptom scales, such as the WOMAC scale, and easy-to-administer scales, such as the pain numeric rating scale in one study. The longitudinal design of this study facilitated the assessment of the relationship between DM and disability over time. The WOMAC total score and its subscales provide a comprehensive assessment of symptoms and physical function, capturing the multidimensional aspects of symptoms and disability. The inclusion of various potential confounding factors, such as age, sex, race, educational level, BMI, depressive symptoms, and physical activity, helps minimize potential confounding effects and enhances the robustness of our findings.

Although our study provides important insights into the relationship between DM and knee OA using a rich OAI dataset, several limitations should be considered. First, our study’s reliance on self-reported DM status is considered a limiting factor. Although comprehensive, the OAI dataset did not include clinically verified DM diagnoses or detailed information on the management and duration of DM. This lack of clinical validation could lead to misclassification biases, as some participants might have undiagnosed DM, or their DM severity may not be accurately represented. Furthermore, the absence of data on glycemic control, such as HbA1c levels, limited our ability to evaluate the relationship between DM control and knee OA progression. Future research should examine this association using objective measures for DM such as fasting plasma glucose and HgA1c and link these measures with pain outcomes longitudinally. Second, the potential for residual confounding remains despite the longitudinal design and extensive range of covariates adjusted in the analysis. Although robust, the OAI dataset may not capture all the relevant variables that could influence the relationship between DM and knee OA. Factors such as specific DM and OA medications, dietary habits, genetic predispositions, and other lifestyle factors could have a significant impact, but these were not completely accounted for in our analysis. Furthermore, the findings of this study, based on a large OAI cohort, may have limited generalizability to broader populations. The OAI cohort predominantly included individuals at high risk of or with existing knee OA, which may not represent the entire spectrum of OA severity or location. The demographic characteristics of the OAI cohort, particularly age, race, and other socioeconomic factors, may limit the applicability of our results to other populations. The results showed that baseline measures differences between knee OA and DM group when compared to knee OA only group indicating an older age, higher BMI, and worse knee symptoms. These baseline imbalances may influence the results. Future research should examine this association using objective measures for DM and glycemic control and the influence of DM duration on these associations with knee OA. The reduction of the sample from 4,796 participants to 2,486 participants is due to the exclusion of those without knee OA (KL < 2) as those without knee OA would affect the observation since they have mild or no pain at early stages (KL < 2). This decrease in sample may affect the generalizability of the results. Finally, this study focused on individuals with knee OA, which limits the generalizability of the findings to other joints (hips and hands).

## Conclusion

This longitudinal cohort study provides evidence supporting the association among baseline DM, increased knee symptoms, and disability observed in participants with knee OA. These findings emphasize the importance of considering comorbid DM in the management and treatment of knee OA. Further research is needed to elucidate the underlying mechanisms driving this association and develop targeted interventions aimed at mitigating disability progression in this high-risk population.

## Data Availability

Publicly available datasets were analyzed in this study. This data can be found at: https://data-archive.nimh.nih.gov/oai/.
